# Are Sexual and Reproductive Behaviors Related to Mental Health Symptoms in Indigenous Communities? A Cross-Sectional Study in La Guajira, Colombia

**DOI:** 10.3390/bs16071236

**Published:** 2026-07-21

**Authors:** Kevin Rico-Gutiérrez, Andrés Felipe Mora-Salamanca, Pedro Barrera-López, Sandra Barrera-Ayala

**Affiliations:** Grupo de Salud Materna y Perinatal, Instituto Nacional de Salud, Avenida Calle 26 No. 51-20—Zona 6 CAN, Bogotá 111321, Colombia; krico@ins.gov.co (K.R.-G.); afmorasa@unal.edu.co (A.F.M.-S.); pbarrera@ins.gov.co (P.B.-L.)

**Keywords:** sexual health, reproductive health, mental health, indigenous peoples, cross-sectional study, Colombia

## Abstract

Sexual and reproductive behaviors and mental health among indigenous peoples are influenced by structural, cultural, and territorial determinants. In Latin America, indigenous populations face inequities related to health service access, sexual education, and mental health care. We assessed the association between sexual and reproductive factors and the presence of mental health symptoms in Wayúu indigenous communities. A cross-sectional study was conducted between 2024 and 2025 in Wayúu communities in Manaure, La Guajira, Colombia. A structured survey including sociodemographic variables, sexual and reproductive history, and mental health symptoms was applied to adult Wayúu indigenous people. A composite score of mental health symptoms was constructed, and the presence of ≥3 symptoms was defined as a “high burden of mental health symptoms.” Descriptive analyses and multivariate logistic regression were performed to estimate adjusted odds ratios (OR). In total, 245 (203 women and 42 men) people participated in the study. The median age was 30 years (IQR 24–38). Forty-nine percent did not receive sex education, and 65% did not use family planning methods. Between 28% and 33% reported persistent symptoms of emotional distress. In multivariable analyses, female sex showed a non-significant association with a higher burden of mental health symptoms (aOR 2.20; 95% CI 0.91–6.19). No statistically significant associations were observed for age, lack of sex education, non-use of family planning methods, or number of children. These findings highlight the high frequency of poor sexual and reproductive health indicators and emotional distress symptoms in Wayúu communities, underscoring the need for integrated, culturally relevant approaches to sexual, reproductive, and mental health care in this population.

## 1. Introduction

Sexual and reproductive health (SRH) is an essential component of the right to health and a key determinant of maternal and perinatal morbidity and mortality worldwide (World Health Organization, 2022). The World Health Organization stated that access to comprehensive sex education, safe contraceptive methods, and culturally relevant health services is fundamental to ensuring reproductive autonomy and health equity (World Health Organization, 2022).

In Latin America, indigenous populations continue to face profound health inequities. Indigenous women have higher rates of adolescent pregnancy, lower family planning coverage, and greater barriers to accessing health services compared to non-indigenous populations (CEPAL, 2014; Pan American Health Organization, 2021). These inequities are linked to structural determinants such as poverty, territorial exclusion, institutional discrimination, and limited intercultural coordination within health systems (Marmot et al., 2008).

In Colombia, the Wayúu people are the country’s largest indigenous community and are mainly located in the northern region of Colombia, particularly in La Guajira department. The region exhibits critical indicators of food insecurity, limited access to drinking water, and geographical barriers to accessing health services (Ministry of Health and Social Protection of Colombia, 2023). These conditions can influence both reproductive health and emotional and mental well-being. Mental health in indigenous populations has historically been underestimated in public health agendas. Furthermore, the coexistence of cultural barriers and institutional limitations can further reduce access to and utilization of mental health care services (Thakur et al., 2024; Vance et al., 2026; Walters & Simoni, 2002).

Recent evidence from Colombia and Latin America suggests that Indigenous populations experience a disproportionate burden of mental health symptoms, structural vulnerability, and barriers to culturally appropriate healthcare. According to the Colombian National Mental Health Survey (Ministry of Health & Minciencias, 2016), 8.3% of Colombian adults self-identified as Indigenous, and this population exhibited higher rates of poverty (26.6%) and displacement-related violence exposure (17.8%) compared with other population groups; the prevalence of mental health problems was 8.1%, including alcohol-related disorders (16.2%) and anxiety or depressive disorders (7.6%). (Agudelo-Hernández et al., 2023). Additionally, studies conducted in Indigenous communities in La Guajira have highlighted persistent barriers to healthcare access, language discordance, geographic isolation, and limitations in intercultural health services for Wayúu populations (Hinds et al., 2025; Mignone et al., 2023).

The theoretical rationale linking sexual and reproductive health (SRH) factors to mental health symptoms draws on a body of evidence establishing shared structural and psychosocial pathways. Globally, a wide-ranging prevalence of mental ill-health has been observed in adolescents and young adults following key SRH events—including pregnancy, childbirth, and sexually transmitted infections—suggesting that SRH events and mental health outcomes are not independent phenomena (Vanderkruik et al., 2021). A systematic review and meta-analysis by Abajobir et al. (2016) found that the prevalence of perinatal depression is approximately two-fold higher among women with unintended pregnancies compared to those with planned pregnancies, highlighting early childbearing and limited reproductive autonomy as independent psychosocial stressors. More broadly, lack of sex education and limited access to family planning have been identified as indicators of reduced SRH agency—and contexts of reduced agency are consistently associated with higher psychological distress, particularly among women facing intersecting structural disadvantages including poverty, geographic marginalization, and limited institutional support (Abacan & Raphael, 2023; Alexander et al., 2016). Taken together, this evidence supports the hypothesis that sexual and reproductive health factors, such as sex education coverage, family planning use, and age at first childbirth, may potentially contribute to greater mental health symptom burden in contexts of structural inequality such as those experienced by Wayúu communities in La Guajira.

In Latin America, there is limited quantitative evidence on the interaction between reproductive health and mental health symptoms in indigenous communities, particularly among the Wayúu population. Most available studies have focused on descriptive or ethnographic aspects, with limited exploration of epidemiological associations between reproductive health factors and emotional well-being (Mejia et al., 2021). We aimed to assess the association between sexual and reproductive behaviors and mental health symptoms in Wayúu communities located in the municipality of Manaure, La Guajira, Colombia.

## 2. Materials and Methods

### 2.1. Study Design

A cross-sectional analytical study was conducted between 2024 and 2025. We followed the STROBE guideline recommendations for observational studies in order to report on our findings (von Elm et al., 2007).

### 2.2. Context and Population

**Figure 1:**
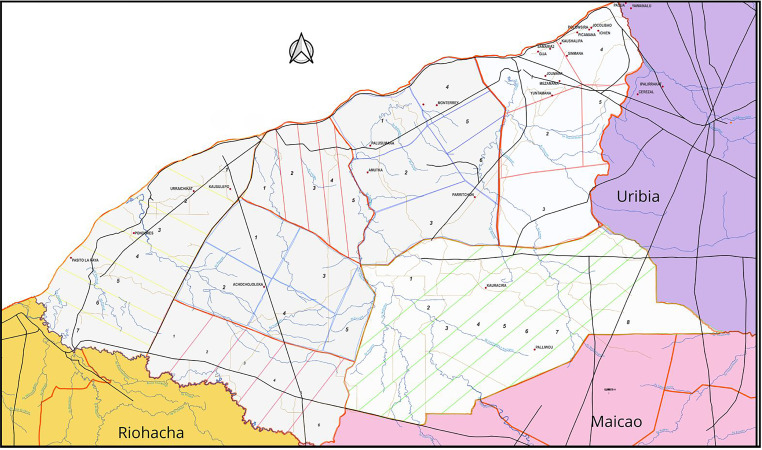
Map of Wayuú Indigenous communities from Manaure, La Guajira, included in the study, showing the high geographic dispersion. Source: Courtesy of Miryis Pushaina Barros based on DANE cartographic data.

According to the 2018 National Population and Housing Census conducted by DANE, the Wayúu are the largest Indigenous people in Colombia, with 380,460 members (Departamento Administrativo Nacional de Estadística [DANE], 2019). In La Guajira, 89.4% of the Wayúu population was concentrated in four municipalities—Uribia (40.7%), Manaure (18.2%), Maicao (17.5%), and Riohacha (13.0%)—with Uribia alone accounting for 40.7% of the Wayúu population in the department (Departamento Administrativo Nacional de Estadística [DANE], 2019, 2021). Although the Wayúu population is primarily concentrated in La Guajira, where 97.5% of the people reside, previous census-based characterizations also describe their presence in neighboring Colombian departments such as Cesar and Magdalena, reflecting a broader regional distribution across northern Colombia (Departamento Administrativo Nacional de Estadística [DANE], 2021). In addition, the Wayúu are a transboundary Indigenous people whose ancestral territory extends across the Guajira Peninsula into Zulia State, Venezuela (Ochoa Sierra, 2026). Men and women from Wayúu indigenous communities in the municipality of Manaure (department of La Guajira, Colombia) were included. Manaure is a territory characterized by high geographic dispersion, difficulties in accessing health services, and persistent conditions of social vulnerability (Departamento Administrativo Nacional de Estadística [DANE], 2019; Hinds et al., 2025) (Figure 1).

### 2.3. Sample Size and Sampling

Sample size estimation was based on an expected proportion of 24% of people that had received sexual and reproductive health education (based on a pilot study conducted on 25 individuals of Wayúu communities prior to the study). A 95% confidence level, and a design effect of 5.0. Under these assumptions, the estimated sample size varied according to the desired precision: 274 participants for 10% precision, 139 participants for 15% precision, and 82 participants for 20% precision. A target of 245 participants was established as a feasible and methodologically justified recruitment goal given the 15% precision estimate and the geographic and logistical constraints of fieldwork in dispersed rural Wayúu communities. Inclusion criteria were adult (≥18 years) Wayúu members and willingness to complete the survey.

Participants were recruited during community outreach visits (field visits) conducted in 23 Wayúu communities in Manaure, La Guajira, Colombia. Additional participants were recruited from Indigenous Health Provider Institutions (IPSI, Instituciones Prestadoras de Salud Indígenas) and from the local public hospital network (ESE, Empresa Social del Estado). A non-probabilistic convenience sampling strategy was used based on accessibility to participating communities (n = 151) and healthcare institutions (n = 94) during fieldwork activities.

### 2.4. Participants

Men and women from Wayúu communities who were present at the time of the visit and voluntarily agreed to participate in the study were included. The survey was created by the authors based on the last Colombian National Mental Health Survey (2015) and relevant variables related to reproductive health in theoretical models.

### 2.5. Variables

#### 2.5.1. Sociodemographic Variables

- Age
- Sex
- Marital status
- Number of household members
- Occupation

#### 2.5.2. Reproductive Variables

- Sex education

Use of family planning methods (current use at the time of the survey, i.e., using any contraceptive method at the time the questionnaire was administered).

- Type of contraceptive method
- Age at first child
- Number of children
- Number of abortions

#### 2.5.3. Mental Health Variables

We operationalized mental health using observable symptoms as proxies of psychosocial distress because nonspecific distress has been established as a clinically meaningful marker of mental health burden in community and low-resource settings (Kessler et al., 2002; Patel & Kleinman, 2003), and because common manifestations such as insomnia, crying, and loss of appetite can be assessed feasibly in routine settings—as evidenced by their inclusion in validated community screening tools for common mental disorders in low- and middle-income countries (Scholte et al., 2011)—while still requiring clinical exclusion of medical causes.

Self-reported symptoms were assessed:

- Poor sleep
- Frequent crying
- Difficulty thinking clearly
- Poor appetite
- Suicidal ideation

### 2.6. Primary Outcome

A composite score of mental symptoms was constructed by summing affirmative responses. The five items included were selected based on the Colombian National Mental Health Survey (ENSM-2015) and are consistent with somatic and affective symptom domains commonly assessed in community mental health screening in low-resource settings (poor sleep, poor appetite, frequent crying, difficulty thinking clearly, and suicidal ideation).

“High burden of mental symptoms” was defined as the presence of three or more symptoms. This threshold was chosen pragmatically to identify individuals with a clinically relevant cluster of concurrent symptoms, consistent with approaches used in similar community-based studies in indigenous populations (Agudelo-Hernández et al., 2023).

### 2.7. Data Collection

Information was collected via a structured survey administered by trained staff. Wayuú translators and interpreters assisted with data collection to help participants who did not speak Spanish understand the survey. Data cleaning and variable standardization were performed prior to analysis.

### 2.8. Biases

To reduce information bias, structured questions and standardized training of interviewers were used. Given the cross-sectional design, it was not possible to establish a temporal relationship between exposures and outcomes.

### 2.9. Statistical Analysis

Continuous variables were described using medians and interquartile ranges (IQR). Categorical variables were expressed as frequencies and percentages.

Bivariate comparisons were performed using the chi-square test or Fisher’s exact test for categorical variables and the Wilcoxon test for continuous variables.

Crude odds ratios were estimated using univariable logistic regression models. Subsequently, a multivariate logistic regression model was constructed to estimate adjusted odds ratios (aOR) and 95% confidence intervals (95% CI) for factors associated with a high burden of mental symptoms.

The following covariates were selected a priori for inclusion in the multivariable model based on theoretical relevance and prior literature: age, sex, use of family planning methods, receipt of sex education, and number of children. These variables were identified as plausible determinants of mental health symptom burden within the conceptual framework underlying this study.

Missing data were assessed for all variables included in the descriptive and multivariable analyses. No imputation was performed. Descriptive analyses were conducted using available data for each variable. For the multivariable logistic regression model, complete-case analysis was used, including only participants with non-missing data for the outcome and all covariates included in the final model.

Analyses were performed using R version 4.4.

### 2.10. Ethical Considerations

The study was conducted in accordance with the Declaration of Helsinki and approved by the Institutional Ethics and Research Methodology Committee (CEMIN 19-2023, Act No. 21, 14 July 2023). The study was agreed upon with traditional Wayúu authorities. All participants provided informed consent. Confidentiality and anonymization of data were ensured.

## 3. Results

### 3.1. Sociodemographic Characteristics

**Figure 2:**
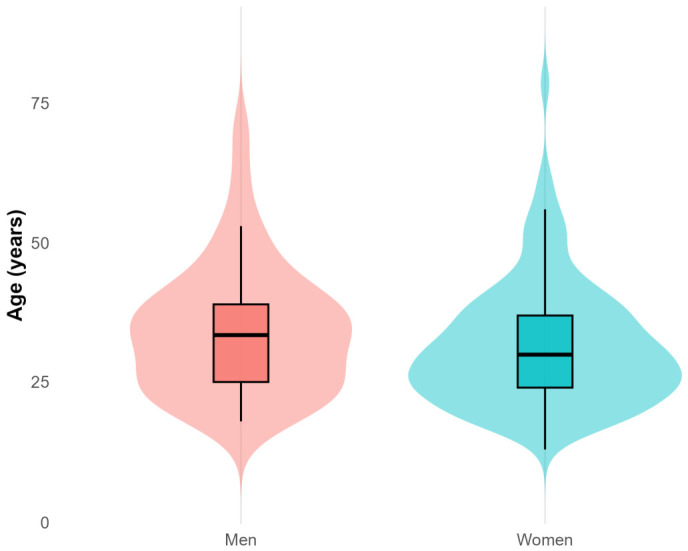
Age distribution by sex.

In total, 245 members of the Wayúu community participated in the study (Table 1). The participant ratio was approximately 5 women to every man. The median age was 30 years (IQR 24–38) (Figure 2). Approximately 70% of participants were cohabiting or married. The median number of women per household was 3 (IQR 2–4) and that of men was 2 (IQR 1–3).

The most common occupations were housework, informal commercial activities, and community work (Table 1).

### 3.2. Sexual and Reproductive Health

**Figure 3:**
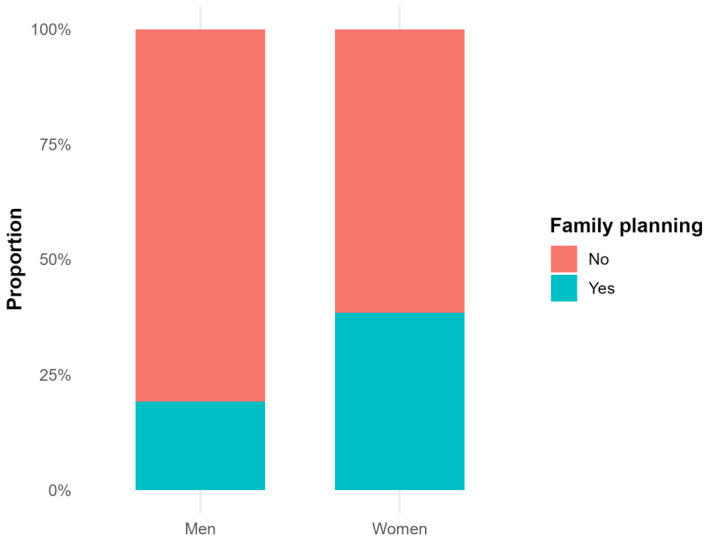
Use of family planning methods.

Of the participants, 49% reported not having received sex education (Table 2). The absence of sex education was more common among men (Figure 3).

**Figure 4:**
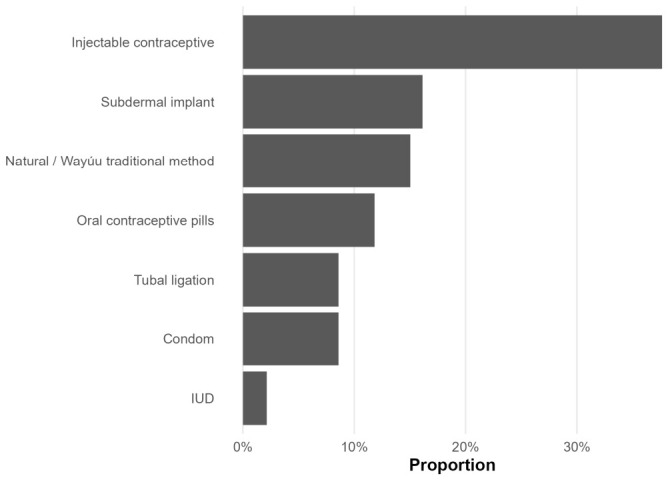
Distribution of contraceptive methods.

Only 35% used some form of family planning. Among women, the most common methods were injectable contraceptives, subcutaneous implants, and traditional methods (Figure 4).

The median age at first child was 18 years (IQR 16–21). The median number of children was 3 (IQR 1–4).

### 3.3. Mental Health Symptoms

**Figure 5:**
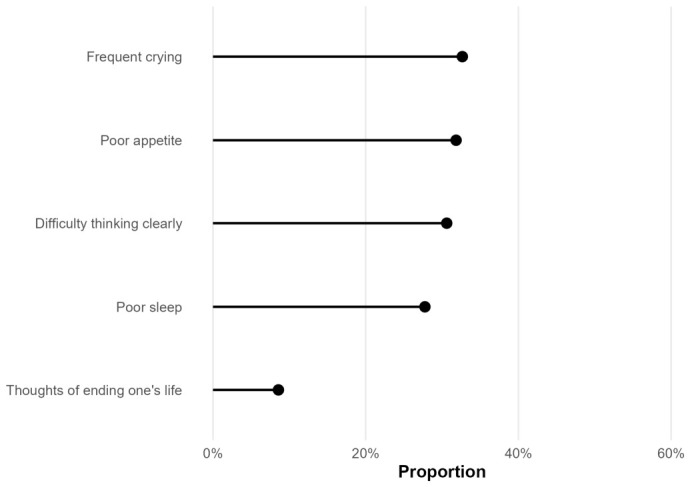
Prevalence of mental health symptoms.

Between 28% and 33% of participants reported persistent symptoms of emotional distress. The most common symptoms were frequent crying, poor appetite, difficulty thinking clearly, and sleep disturbances (Figure 5). Approximately 10% reported suicidal ideation.

### 3.4. Factors Associated with a High Burden of Mental Symptoms

A total of 56 participants (22.9%) presented a high mental symptom burden. Age, gender, family planning use, sex education, and number of children were included in the multivariable logistic regression model (Table 3). Female sex showed a non-significant trend toward higher odds of presenting a high mental symptom burden (aOR 2.20; 95% CI 0.91–6.19; *p* = 0.10). No statistically significant associations were observed for age (aOR 1.02; 95% CI 0.990000–1.05), non-use of family planning methods (aOR 1.11; 95% CI 0.59–2.14), lack of sex education (aOR 1.03; 95% CI 0.56–1.92), or number of children (aOR 0.91; 95% CI 0.78–1.04).

## 4. Discussion

The present study identified a high prevalence of poor indicators of sexual and reproductive behaviors and mental health symptoms in Wayúu communities in Manaure. We did not find that the absence of sex education and the non-use of family planning methods were associated with a higher burden of emotional symptoms.

The limited coverage of sex education observed is consistent with previous reports in Latin American indigenous populations (CEPAL, 2014; Mejia et al., 2021). Geographic barriers, limited availability of culturally relevant services, and structural inequalities continue to affect access to SRH information and services. The young age at first childbirth observed in this study has also been described in vulnerable indigenous contexts and may affect educational trajectories, economic autonomy, and emotional well-being (United Nations, 2010).

The mental health symptom burden observed in this study—with 22.9% of participants meeting the high symptom criterion and 8.6% reporting suicidal ideation—is broadly consistent with, and in some dimensions exceeds, the available evidence from comparable indigenous and rural populations. International studies have shown that indigenous populations may experience higher rates of depression and suicide in contexts of territorial exclusion and social vulnerability (Chandler & Lalonde, 2008; Gone & Trimble, 2012; Kirmayer et al., 2014).

In the Colombian context specifically, the 2015 National Mental Health Survey found that indigenous Colombians reported higher rates of displacement due to violence and mental illness—including anxiety, depression, and substance abuse—than the general population, and that loss of cultural identity, operationalized as not speaking one’s indigenous language and living in urban areas, was independently associated with poorer mental health outcomes. These findings from the ENSM-2015 (Gómez-Restrepo et al., 2016) provide a national reference point against which the Wayúu data can be interpreted: the mental health burden identified in the present study in a rural, linguistically intact indigenous community suggests that structural vulnerability—rather than cultural displacement alone—constitutes a significant independent driver of psychosocial distress.

At the regional level, indigenous peoples of Latin America remain severely excluded from mainstream society and from mental health services specifically designed for them, with the lack of research on the subject meaning that comparative statistics are virtually unavailable (Incayawar & Maldonado-Bouchard, 2009). This evidential gap reinforces the descriptive and contextual value of the present study, even in the absence of statistically significant associations in the multivariable model. Furthermore, a systematic review and meta-analysis of studies comparing mental health disorder prevalence in indigenous populations of the Americas with non-indigenous controls found that results were generally similar across sub-analyses of Latin America, Canada, and the United States, with risk factors for psychiatric illness likely representing a complex interaction of biological, educational, economic, and sociocultural factors that vary between disorders (Kisely et al., 2016)—a conclusion directly applicable to the Wayúu context, where the intersection of territorial vulnerability, limited institutional access, and cultural disruption cannot be disentangled from the observed symptom burden.

Although no statistically significant association was identified between indicators of reproductive vulnerability and mental health symptom burden, the descriptive findings reveal a population facing concurrent and substantial challenges in both domains. The high prevalence of limited sex education (49%), low family planning coverage (35%), and mental health symptoms (22.9% with high symptom burden) may reflect shared underlying structural determinants—including geographic isolation, institutional barriers, and cultural discontinuity—rather than a direct causal pathway between reproductive and mental health outcomes.

Several methodological limitations must be acknowledged when interpreting these findings. The use of convenience sampling, the pronounced gender imbalance (83% women), and the partial recruitment of participants through health care institutions may limit the representativeness of the sample and introduce selection bias toward individuals with greater institutional contact. The underrepresentation of men (n = 42) is likely attributable to the timing of community visits, which coincided with periods when male community members were engaged in work activities outside the ranchería; however, this imbalance constrains the generalizability of findings to the male Wayúu population and substantially limits the statistical power of gender-stratified comparisons.

Furthermore, with only 56 participants meeting the high mental health symptom burden criterion, the study was underpowered to detect associations of moderate effect size between reproductive vulnerability indicators and mental health outcomes. In this context, the absence of statistically significant associations in the multivariable model should not be interpreted as evidence that no relationship exists; rather, a type II error—that is, the failure to detect a true effect due to insufficient sample size—cannot be excluded. Variables not included in the model (e.g., current pregnancy status, history of intimate partner violence) were not systematically assessed in all participants and could not be reliably incorporated. The absence of a validated psychometric instrument adapted for the Wayúu population is also acknowledged as a limitation of this study.

For the Wayuú context specifically, evidence-informed recommendations include: (1) incorporating the palabrero (traditional conflict mediator) and traditional midwives as co-facilitators in sexual health and mental health programs; (2) developing bilingual (Wayuunaiki–Spanish) educational materials on family planning that integrate both biomedical and traditional Wayuú contraceptive knowledge without establishing hierarchies between systems; and (3) establishing community-based psychosocial support pathways anchored in the matrilineal family structure, given that none of the participants in this study reported seeking formal mental health care. These strategies align with recommendations from intercultural health models implemented in Colombia and other Latin American countries with large indigenous populations.

Future longitudinal studies would allow for the exploration of temporal relationships and causal mechanisms between sexual and reproductive behaviors and mental health in indigenous populations. Our findings highlight the need for comprehensive care models that integrate sexual health and mental health through culturally relevant approaches. Further studies should be designed with larger, gender-balanced samples, probability-based sampling strategies, and validated mental health screening instruments culturally adapted for Wayúu communities—such as culturally adapted versions of the PHQ-9, GAD-7, or the Kessler Psychological Distress Scale—to adequately test the hypothesized associations.

## 5. Conclusions

Low coverage of sex education, non-use of family planning and mental health symptoms were frequent among participants from Wayúu communities in La Guajira. Although no statistically significant associations were identified in multivariable analyses, the observed trends highlight the need for further studies exploring the interaction between sexual and reproductive health and emotional well-being in Indigenous populations.

## Figures and Tables

**Table 1:** Sociodemographic characteristics.

Characteristic	Male N = 42 *	Female N = 203 *	Overall N = 245 *
Age (years)	34 (25, 39)	30 (24, 37)	30 (24, 38)
Marital status			
Married	12 (29%)	36 (18%)	48 (20%)
Separated	2 (4.8%)	15 (7.4%)	17 (6.9%)
Single	7 (17%)	30 (15%)	37 (15%)
Cohabiting	19 (45%)	116 (57%)	135 (55%)
Widow	2 (4.8%)	6 (3.0%)	8 (3.3%)
Occupation			
Artisan	2 (4.8%)	61 (30%)	63 (26%)
Household	0 (0%)	89 (44%)	89 (37%)
Other	40 (95%)	51 (25%)	91 (37%)
(No data)	0	2	2
Number of men per household	2.00 (1.00, 3.00)	2.00 (1.00, 3.00)	2.00 (1.00, 3.00)
Number of women per household	3.00 (1.00, 4.00)	3.00 (2.00, 4.00)	3.00 (2.00, 4.00)

* Median (Q1, Q3); n (%).

**Table 2:** Sexual, Reproductive, and Mental Health.

Characteristic	Men N = 42 *	Women N = 203 *	Overall N = 245 *
Sex education			
No	25 (60%)	96 (47%)	121 (49%)
Yes	17 (40%)	107 (53%)	124 (51%)
Family planning			
No	34 (81%)	125 (62%)	159 (65%)
Yes	8 (19%)	78 (38%)	86 (35%)
What method do you use for birth control?			
Condom	8 (89%)	0 (0%)	8 (8.6%)
IUD	0 (0%)	2 (2.4%)	2 (2.2%)
Subcutaneous implant	0 (0%)	15 (18%)	15 (16%)
Contraceptive injection	0 (0%)	35 (42%)	35 (38%)
Tubal ligation	0 (0%)	8 (9.5%)	8 (8.6%)
Natural method/Wayúu culture	1 (11%)	13 (15%)	14 (15%)
Birth control pills	0 (0%)	11 (13%)	11 (12%)
(No data)	33	119	152
Why are you not planning?			
Wants to get pregnant/have more children	1 (4.2%)	15 (13%)	16 (12%)
Pregnant	0 (0%)	7 (6.3%)	7 (5.1%)
Sterilization	0 (0%)	12 (11%)	12 (8.8%)
Don’t know	3 (13%)	3 (2.7%)	6 (4.4%)
Has not had sexual intercourse	0 (0%)	1 (0.9%)	1 (0.7%)
Does not like it/side effects	7 (29%)	16 (14%)	23 (17%)
Other	10 (42%)	42 (38%)	52 (38%)
By age	2 (8.3%)	2 (1.8%)	4 (2.9%)
Single/partner absent	1 (4.2%)	11 (9.8%)	12 (8.8%)
Already using a method	0 (0%)	3 (2.7%)	3 (2.2%)
(No data)	18	91	109
At what age did you get your first period? (Years)	NA (NA, NA)	13.00 (12.00, 14.00)	13.00 (12.00, 14.00)
(No data)	42	0	42
How many children have you had?	2.00 (1.00, 4.00)	3.00 (1.00, 4.00)	3.00 (1.00, 4.00)
(No data)	0	2	2
How many living children do you have?	2.00 (1.00, 4.00)	3.00 (1.00, 4.00)	2.00 (1.00, 4.00)
(No data)	0	1	1
At what age did you have your first child?	20 (17, 23)	18 (16, 21)	19 (16, 22)
(No data)	0	1	1
How many abortions have you had?			
0	0 (NA%)	180 (89%)	180 (89%)
1	0 (NA%)	16 (7.9%)	16 (7.9%)
2	0 (NA%)	4 (2.0%)	4 (2.0%)
3	0 (NA%)	2 (1.0%)	2 (1.0%)
(No data)	42	1	43
Did you sleep poorly?			
No	33 (79%)	144 (71%)	177 (72%)
Yes	9 (21%)	59 (29%)	68 (28%)
Have you had a poor appetite?			
No	31 (74%)	136 (67%)	167 (68%)
Yes	11 (26%)	67 (33%)	78 (32%)
Have you cried frequently?			
No	34 (81%)	131 (65%)	165 (67%)
Yes	8 (19%)	72 (35%)	80 (33%)
Have you felt like you couldn’t think clearly?			
No	37 (88%)	133 (66%)	170 (69%)
Yes	5 (12%)	70 (34%)	75 (31%)
Have you ever thought about ending your life?			
No	37 (88%)	187 (92%)	224 (91%)
Yes	5 (12%)	16 (7.9%)	21 (8.6%)
High mental load			
No	36 (86%)	153 (75%)	189 (77%)
Yes	6 (14%)	50 (25%)	56 (23%)

* n (%); Median (Q1, Q3).

**Table 3:** Factors associated with a high burden of mental symptoms.

Characteristic	OR (95% CI)	*p*-Value	aOR (95% CI)	*p*-Value
Age (years)	1 (0.98, 1.03)	0.76	1.02 (0.99, 1.05)	0.27
Gender				
Male	—	—	—	—
Female	1.99 (0.84, 5.49)	0.14	2.20 (0.91, 6.19)	0.10
Family planning use				
Yes	—	—	—	—
No	1.06 (0.57, 2.02)	0.85	1.11 (0.59, 2.14)	0.74
Sex education				
Yes	—	—	—	—
No	0.92 (0.51, 1.68)	0.79	1.03 (0.56, 1.92)	0.92
Number of children	0.95 (0.84, 1.06)	0.40	0.91 (0.78, 1.04)	0.19

Abbreviations: CI = Confidence Interval, OR = Odds Ratio.

## Data Availability

The data presented in this study are available upon request from the corresponding author due to legal reasons.

## Abbreviations

The following abbreviations are used in this manuscript:

aOR	Adjusted Odds Ratio
ASIS	Análisis de Situación de Salud
CEPAL	Comisión Económica para América Latina y el Caribe
CI	Confidence Interval
DANE	Departamento Administrativo Nacional de Estadística
IQR	Interquartile Range
IUD	Intrauterine Device
MSPS	Ministerio de Salud y Protección Social
NA	Not Available/Not Applicable
OR	Odds Ratio
PAHO	Pan American Health Organization
SRH	Sexual and Reproductive Health
STROBE	Strengthening the Reporting of Observational Studies in Epidemiology
WHO	World Health Organization
